# Identification of rogue datasets in serial crystallography^1^

**DOI:** 10.1107/S1600576716005471

**Published:** 2016-04-18

**Authors:** Greta Assmann, Wolfgang Brehm, Kay Diederichs

**Affiliations:** a Department of Biology, University of Konstanz, Box 647, Konstanz, D-78457, Germany

**Keywords:** serial crystallography, outlier identification, CC_1/2_, precision, model bias, isomorphism, non-isomorphism

## Abstract

Advances in beamline optics, detectors and X-ray sources allow new techniques of crystallographic data collection. In serial crystallography, a large number of partial datasets from crystals of small volume are measured. Merging of datasets from different crystals in order to enhance data completeness and accuracy is only valid if the crystals are isomorphous, *i.e.* sufficiently similar in cell parameters, unit-cell contents and molecular structure. Identification and exclusion of non-isomorphous datasets is therefore indispensable and must be done by means of suitable indicators. To identify rogue datasets, the influence of each dataset on CC_1/2_ [Karplus & Diederichs (2012 ▸). *Science*, **336**, 1030–1033], the correlation coefficient between pairs of intensities averaged in two randomly assigned subsets of observations, is evaluated. The presented method employs a precise calculation of CC_1/2_ that avoids the random assignment, and instead of using an overall CC_1/2_, an average over resolution shells is employed to obtain sensible results. The selection procedure was verified by measuring the correlation of observed (merged) intensities and intensities calculated from a model. It is found that inclusion and merging of non-isomorphous datasets may bias the refined model towards those datasets, and measures to reduce this effect are suggested.

## Abstract 2

In serial crystallography, CC_1/2_ may be used as an optimization target, and outlier datasets can be identified on the basis of their influence on the average CC_1/2_ of the merged data. This leads to the ΔCC_1/2_ method presented here.

## 1. Introduction   

Several bottlenecks hamper structure determination of biological macromolecules. One practical problem is often the lack of suitably large crystals for collection of complete high-resolution data, as the diffraction signal for a given incident X-ray beam is proportional to the well ordered crystal volume illuminated by the beam, as given by Darwin’s formula (Darwin, 1914 ▸, 1922 ▸; Blundell & Johnson, 1976 ▸). Smaller crystals require higher X-ray beam intensities to produce diffraction up to the same resolution as comparable crystals with larger volume, but because of radiation damage do not result in complete datasets.

This problem of incompleteness has been addressed by combining several partial datasets from multiple crystals in order to obtain a complete dataset averaged (‘merged’) over all observations of every unique reflection. Merging of data from a few (2–20) crystals has been standard practice in crystallography (Kendrew *et al.*, 1960 ▸; Dickerson *et al.*, 1961 ▸), but recently this concept was extended to tens or even thousands of crystals with only a few reflections per dataset and termed serial crystallography (SX). When using extremely short pulses of X-rays, each of tens of femtoseconds in duration, generated by a free-electron laser (FEL) the method is referred to as serial femtosecond crystallography (SFX; Chapman *et al.*, 2011 ▸). This also exploits the ‘diffraction before destruction’ approach, where single diffraction ‘snapshots’ are collected before radiation damage can occur. Serial crystallography performed at the synchrotron (SSX; Rossmann, 2014 ▸) is a more recent development based on data collection and processing methods established for single-crystal work, but enhanced by procedures for crystal identification by scanning crystallization plates using conventional optics or X-rays. This approach is also ideally suited for employing novel crystallization setups such as the lipidic cubic phase and *in situ* data collection at room or cryo temperature (Huang *et al.*, 2015 ▸, 2016 ▸).

Although serial crystallography, especially SFX, can in principle solve or mitigate the problem of radiation damage and the lack of sufficiently large crystals, one potential obstacle is non-isomorphism of crystals. In order to merge different partial datasets into one complete dataset, one must ensure that all partial datasets are sufficiently isomorphous. Isomorphous crystals are structurally identical and correspond to the same atomic model such that datasets only differ by random error, whereas non-isomorphous crystals represent different structural features, at the level of cell parameters, composition (in terms of presence of molecular entities like ligands and solvent molecules) or molecular conformation. The variation between datasets thus also depends on the extent of non-isomorphism.

To group datasets on the basis of similarity, hierarchical clustering based on pairwise correlation coefficients was employed by Giordano *et al.* (2012 ▸). The basic idea here is that a low correlation coefficient indicates unrelatedness of datasets, which is interpreted as non-isomorphism. This method may also falsely reject datasets with a high level of random error, or in other words, weak datasets. Essentially, this would trade accuracy for precision.

Another method that can be employed is hierarchical clustering of datasets based on their cell parameters (Foadi *et al.*, 2013 ▸). This method avoids the problem of false positive rejection of weak datasets, but on the other hand similarity of cell parameters is only a necessary but not a sufficient condition, and does not take similarity of diffraction into account. The criterion can therefore be considered as a rather weak filter.

Yet another approach combines the unit-cell changes, the intensity correlation coefficient and a relative anomalous correlation coefficient for clustering of datasets (Liu *et al.*, 2013 ▸).

Following previous work (Karplus & Diederichs, 2012 ▸; Diederichs & Karplus, 2013 ▸), we chose the numerical value of CC_1/2_, an indicator for the precision of the data resulting from merging of the partial datasets, as an optimization target. We have shown earlier that CC_1/2_ limits – as seen from the properties of the derived quantity CC* – the ability to obtain good agreement between model and data (Karplus & Diederichs, 2012 ▸). Since the goal of structure analysis is to obtain a model consistent with the data, it appears logical to optimize CC_1/2_.

CC_1/2_ can be evaluated and optimized as a function of the datasets being merged, and we propose and study a procedure to identify non-isomorphous crystals from multi-crystal datasets based on their influence on CC_1/2_. Simulated data as well as experimental datasets from two projects, PepT and AlgE (Huang *et al.*, 2015 ▸), were analysed to identify non-isomorphous datasets.

## 2. Methods   

### 2.1. Simulated data   

Eleven complete datasets to 1.46 Å resolution were simulated with* SIM_MX* (Diederichs, 2009 ▸) using the atomic coordinates of cubic insulin [Protein Data Bank (PDB) code 2bn3 (Nanao *et al.*, 2005 ▸); space group *I*2_1_3] and a flat bulk solvent (density 0.35 e^−^ A^−3^ and *B* = 50 Å^2^) for the calculation of structure factors. To simulate a specific case of non-isomorphism by numerical experiments, the unit-cell parameters were elongated by different amounts (0–1 Å). This results in a different sampling of the molecular transform and thus changes the intensities. Artificial *F*_calc_^2^ to be used as intensities were then calculated using *PHENIX.FMODEL* (Adams *et al.*, 2002 ▸), and simulated raw datasets were calculated with *SIM_MX* and processed with *XDS* (Kabsch, 2010*a* ▸,*b* ▸). Different random seeds ensured that different (pseudo-) random errors for different datasets were calculated.

### 2.2. Experimental data   

Crystallization and data collection of the membrane protein peptide transporter PepT from *Streptococcus thermophilus* (483 residues) and AlgE, the alginate transporter from *Pseudomonas aeruginosa* (490 residues), were described previously (Huang *et al.*, 2015 ▸). PepT and AlgE X-ray diffraction data consisting of small rotation wedges of different crystals, to 2.78 and 2.54 Å resolution, respectively, were collected at room temperature from crystals *in meso* and *in situ* at the PX II beamline of the Swiss Light Source (Villigen, Switzerland). Each rotation wedge was collected from a different crystal and is denoted as a (partial) dataset in the following. Data were processed with *XDS* and *XSCALE* (Kabsch, 2010*a* ▸,*b* ▸). For this work, not all datasets that were used at the time of PDB deposition were available; thus for PepT, we used 159, and for AlgE, we used all 266 datasets available, 175 of which were used in the work of Huang *et al.* (2015 ▸). Table 1 ▸ summarizes these data.

### 2.3. Calculation of CC_1/2_: the σ–τ method   

For the calculations of CC_1/2_ the *XSCALE* output file (XSCALE.HKL) was used. Merged intensities of observations were weighted with their sigma values as assigned by the data processing programs, *XDS* and *XSCALE*.

As defined by Karplus & Diederichs (2012 ▸), CC_1/2_ can be calculated from the formula for Pearson’s correlation coefficient: 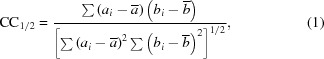
where *a_i_* and *b_i_* are the intensities of unique reflections merged across the observations randomly assigned to subsets A and B, respectively, and 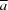 and 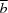 are their averages.

For this work, we calculated CC_1/2_ in a different way, avoiding the random assignment to subsets. CC_1/2_ may be expressed as (Karplus & Diederichs, 2012 ▸, supplement) 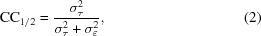
with

τ = difference between true values of intensities and their average (thus τ has zero mean),

∊_A,B_ = random errors in merged intensities of half-sized subsets (with zero mean) which are mutually independent and on average of equal magnitude,


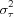
 = variance of τ,


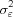
 = variance of 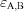.

Then, the full dataset merged intensity *y* (after subtraction of the average) is 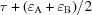 and 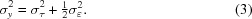



We may thus substitute 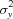 − 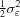 for 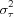 and obtain 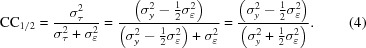
This just requires calculation of 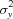, the variance of the average intensities across the unique reflections of a resolution shell, and 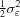, the average variance of the observations contributing to the merged intensities.

As shown above, this ‘σ–τ method’ of CC_1/2_ calculation is mathematically equivalent to the calculation of a Pearson correlation coefficient for the special case of two sets of values (intensities) that randomly deviate from their common ‘true’ values. Since it avoids the random assignment into half-datasets, it is not influenced by any specific random number sequence and thus yields more consistent values, as further discussed below (§4.1).

The average intensity of macromolecular diffraction data diminishes with increasing resolution. If all data, from low to high resolution, are used for calculation of an overall CC_1/2_, the resulting correlation coefficient is dominated by the strong low-resolution terms and therefore biased towards large positive values; the overall CC_1/2_ is thus almost meaningless (Karplus & Diederichs, 2015 ▸). More meaningful CC_1/2_ values are obtained when dividing the data into (usually ten or more) resolution shells, since in each resolution shell the average intensity can be considered constant. To obtain a single value, we average the CC_1/2_ values obtained in all resolution shells, while weighting with the number of contributing reflections.

### 2.4. The ΔCC_1/2_ method   

Since our goal is to maximize CC_1/2_ by excluding datasets, we used the following simple algorithm that avoids any combinatorial explosion of possibilities. We define as CC_1/2_overall_ the value of the average CC_1/2_ when all datasets are included for calculation. Furthermore, we denote as CC_1/2_*i*_ the value of CC_1/2_ when all datasets are included except for one dataset *i*, which is omitted from calculations. CC_1/2_*i*_ is calculated for every dataset *i*. We define 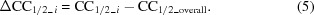
If ΔCC_1/2_*i*_ > 0 (< 0), this dataset is improving (impairing) the overall CC_1/2_. After rejection of the dataset belonging to the worst negative ΔCC_1/2_*i*_, all remaining datasets are processed by *XSCALE* again, because any dataset influences all scale factors. The newly scaled output file can be used to identify another potential non-isomorphous dataset, and this may be iterated until no further significant improvement is obtained; usually, two or three iterations are sufficient.

### 2.5. Validation of isomorphous dataset selection   

We devised a strategy to assess an actual improvement of the data by comparison with the squared *F*_calc_ moduli obtained from a structural model. To this end, deposited PDB models (4xnj for PepT, 4xnk for AlgE; Huang *et al.*, 2015 ▸) were processed with *PHENIX.FMODEL* to produce *F*_calc_^2^ reference data, to be used for comparison with *I*_obs_. We define CC_FOC_overall_ as the correlation coefficient of observed and calculated intensities when all datasets are included. Moreover, we define CC_FOC_*i*_ as the correlation coefficient with all datasets included except for one dataset *i*, which is omitted from calculations. The difference 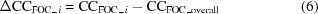
then gives the improvement or impairment the dataset *i* causes in the overall correlation of data and model. ΔCC_FOC_*i*_ < 0 indicates an impairment in the similarity of data and model; ΔCC_FOC_*i*_ > 0 indicates an improvement. We chose to compare ΔCC_FOC_*i*_ and ΔCC_1/2_*i*_ although it would be more appropriate to use 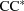 (Karplus & Diederichs, 2012 ▸) instead of CC_1/2_, or in other words, to compare ΔCC_FOC_*i*_ and a quantity defined analogously to ΔCC_1/2_*i*_, Δ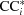 = 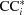 − 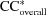. However, since 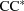 depends monotonically on CC_1/2_, any qualitative finding obtained in a comparison of ΔCC_FOC_*i*_ and ΔCC_1/2_*i*_ would be the same as for a ΔCC_FOC_*i*_ and Δ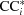 comparison.

For some calculations, random shifts of atom coordinates of the original PDB file were introduced by *MOLEMAN2* (Kleywegt, 1995 ▸).

## 3. Results   

### 3.1. Characteristics of ΔCC_1/2_ for the simulated data   

Eleven complete datasets with different changes in the unit-cell parameters were used to simulate a realistic case where most of the datasets are isomorphous relative to each other but some are (non-isomorphous) outliers.

The ΔCC_1/2_ method was applied to the simulated datasets. In general, increasing changes in unit-cell parameters are associated with decreasing ΔCC_1/2_*i*_, as expected (Table 2 ▸). The largest change in unit-cell parameters (1.0 Å) shows the lowest ΔCC_1/2_*i*_, which is thus correctly identifying non-isomorphism. ΔCC_1/2_*i*_ shows highly positive values for those datasets where no or only slight changes were introduced (0.0–0.2 Å). ΔCC_1/2_*i*_ does not increase linearly; rather, it drops dramatically from 0.2 to 0.4 Å. On the basis of ΔCC_1/2_*i*_, the most isomorphous dataset is the one with a 0.2 Å cell parameter change; this appears to be a sensible result since its intensities are the most closely related to those of all other datasets.

The numerical values of ΔCC_1/2_*i*_ change to a much greater extent (−0.5 to 0.8) than in the experimental case. This is because the impact of one complete dataset (out of 11) is high in comparison to a single small rotation wedge SSX dataset (out of hundreds). Moreover, the artificially induced gap between nearly perfect isomorphous datasets (cell changes of 0.0–0.4 Å, *i.e.* close to the average of all cell changes) and very few severely non-isomorphous datasets (cell changes of 0.6–1.0 Å) enforces this effect.

### 3.2. PepT: model unbiased by non-isomorphous dataset   

**Figure 1:**
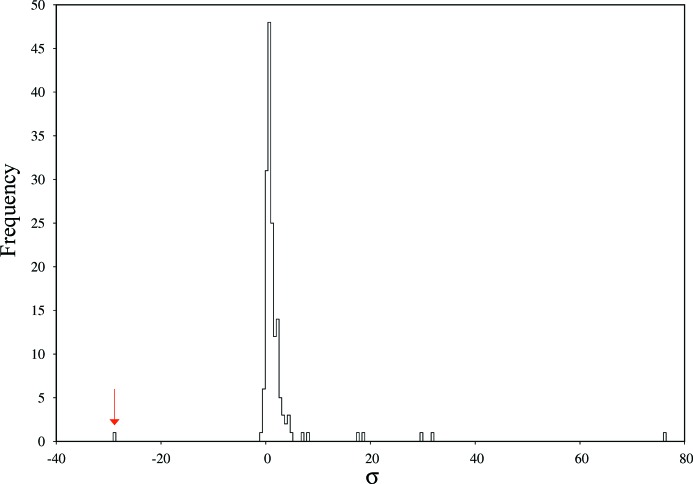
Histogram of ΔCC_1/2_*i*_ values for PepT. The −28.8σ unit outlier is indicated with an arrow.

For PepT, 159 datasets were analysed, as seen in Fig. 1 ▸, where a histogram of ΔCC_1/2_*i*_ is shown. The histogram is dominated by a Gaussian-shaped central part slightly above ΔCC_1/2_ = 0, with standard deviation σ = 1.68 × 10^−4^. Datasets with a ΔCC_1/2_*i*_ of around zero do not significantly change the overall CC_1/2_; however, their inclusion is necessary for increased completeness and multiplicity. One dataset has a significantly (−28.8σ) negative ΔCC_1/2_*i*_ and is thus identified as non-isomorphous. Some datasets have, at 31.8σ and 76.2σ, a highly positive ΔCC_1/2_*i*_; they significantly decrease CC_1/2_overall_ if rejected and appear to be particularly valuable.

Comparing, in terms of raw data appearance and processing logfiles and statistics reported by *XDS* or *XSCALE*, positive or negative outlier datasets with datasets from the central part of the histogram showed no striking peculiarities for any of the analysed criteria such as number of reflections or similar. In particular, negative ΔCC_1/2_*i*_ was not predictable from unit-cell parameters, spot shape or other data processing statistics of single datasets. Furthermore, the negative outlier had not been identified by the ISa-based (Diederichs, 2009 ▸) rejection of datasets performed by Huang *et al.* (2015 ▸). However, we note that important experimental variables, like crystal volume, are not recorded during the experiments.

**Figure 2:**
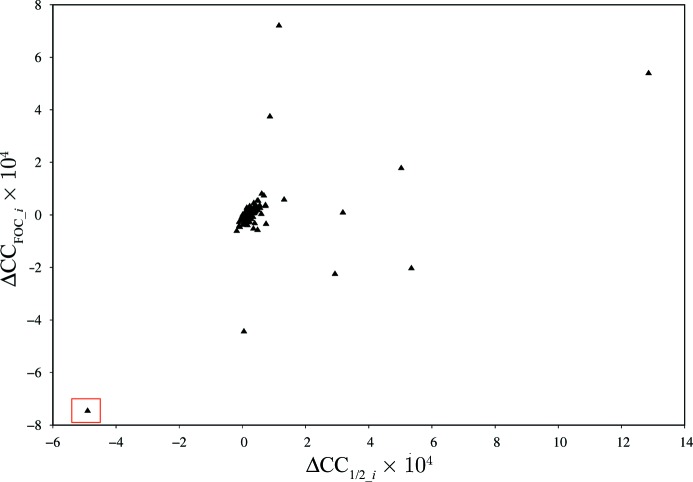
Plot of ΔCC_FOC_*i*_ against ΔCC_1/2_*i*_ for PepT. The −28.8 8σ unit outlier (ΔCC_1/2_*i*_ ≃ −4.8 × 10^−4^) is boxed.

Fig. 2 ▸ shows a plot of ΔCC_FOC_*i*_ against ΔCC_1/2_*i*_ for the datasets of this project. If our procedure for identifying non-isomorphism is meaningful, we expect an improvement of the correlation between model *F*_calc_^2^ and merged *I*_obs_ for those datasets that increase CC_1/2_, and a decrease of correlation for the non-isomorphous ones. As could be expected from the histogram of Fig.1 ▸, most of the data sets cause little change of ΔCC_FOC_ and ΔCC_1/2_; they cluster in the middle of the diagram. The dataset identified as the most non-isomorphous one indeed shows the worst correlation of experimental data and model; CC_FOC_ is significantly improved when rejecting this specific dataset. Conversely, some of the datasets show a clear improvement of CC_FOC_ and CC_1/2_.

The validation appears to work satisfactorily despite the fact that the 4xnj model derived from cryo data and used here for validation is itself not isomorphous with the data, since the cell parameters of the cryo and the room-temperature crystals differ in *a* and *b* by about 4%.

### 3.3. AlgE: model biased by non-isomorphous dataset   

**Figure 3:**
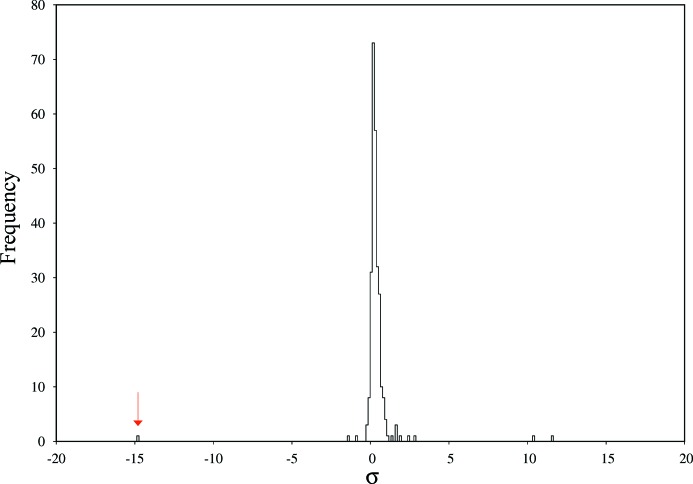
Histogram of ΔCC_1/2_*i*_ values for AlgE. The −14.8σ unit outlier is indicated with an arrow.

AlgE displays a similar histogram (σ = 7.22 × 10^−4^) of ΔCC_1/2_*i*_ values (Fig. 3 ▸) as PepT. The worst non-isomorphous dataset is, at −14.8σ units, an obvious outlier of the ΔCC_1/2_*i*_ distribution. Similarly, positive outliers exist at 10.1σ and 11.5σ units. As for PepT, we did not observe in the raw data or in the processing and scaling statistics any particular properties of positive or negative outliers. Again, the strongest negative outlier had not been identified by the ISa-based rejection of datasets performed by Huang *et al.* (2015 ▸).

**Figure 4:**
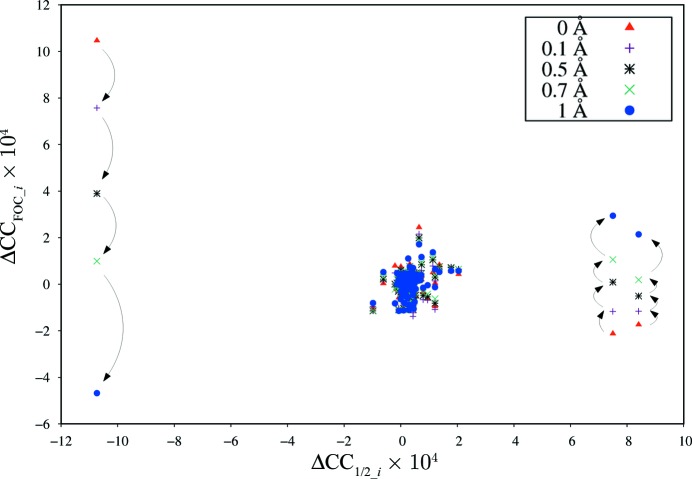
Plot of ΔCC_FOC_*i*_ against ΔCC_1/2_*i*_ for AlgE. Different colours and marker symbols refer to the different random shifts of the atom coordinates. Arrows indicate the change of ΔCC_FOC_*i*_ upon increasing the magnitude of random shifts for the three most significant outliers of the Gaussian distribution of Fig. 3 ▸.

In contrast to Fig. 2 ▸, the plot of ΔCC_FOC_*i*_ against ΔCC_1/2_*i*_ for AlgE shows an unexpected behaviour (Fig. 4 ▸). As expected, most of the datasets cluster at ΔCC_1/2_ and ΔCC_FOC_ values around zero, but the dataset identified by ΔCC_1/2_*i*_ as the most non-isomorphous dataset is surprisingly showing the best ΔCC_FOC_*i*_. Conversely, the best datasets as judged from ΔCC_1/2_*i*_ exhibit negative ΔCC_FOC_*i*_.

After some investigation, we attributed these observations to our choice of PDB models used for validation. In the case of PepT, we had chosen the model based on an independent single-crystal cryo dataset (4xnj), whereas in the case of AlgE, we had chosen 4xnk which had been refined against those datasets we were now trying to characterize. In the case of AlgE, refinement of the model 4xnk against the SSX data had led to a bias in the sense that the model partly fits the systematic effects contributing to the non-isomorphism of the worst dataset. This bias additionally leads to a reduced ΔCC_FOC_*i*_ since the model is biased into a state more distant from those of datasets with positive ΔCC_1/2_*i*_.

We therefore performed an additional experiment: By applying random shifts of magnitude up to 1.0 Å to the 4xnk coordinates, we attempted to ‘shake’ the model out of its local minimum. Recalculating ΔCC_FOC_*i*_ for increasing shifts, we progressively observed the expected behaviour of a positive correlation between ΔCC_1/2_*i*_ and ΔCC_FOC_*i*_, as emphasized by arrows in Fig. 4 ▸. This is most pronounced for the dataset with largest negative ΔCC_1/2_*i*_, which is seen to have strongly negative ΔCC_FOC_*i*_ when the model is shaken most. However, the datasets with most positive ΔCC_1/2_*i*_ did not fully reach high values of ΔCC_FOC_*i*_, presumably because, for the highest shifts, the model is strongly degraded so that it cannot fit the data well.

Ideally, validation is done with a model that is independent of the data. Another experiment was therefore performed with the 4xnl coordinates derived from a dataset collected at cryo temperature. In this case, no bias is present, and indeed there is a positive correlation between ΔCC_1/2_*i*_ and ΔCC_FOC_*i*_ (data not shown), similar to Fig. 2 ▸ for PepT, as expected.

## 4. Discussion   

### 4.1. Calculation of CC_1/2_   

We have shown above that CC_1/2_ can be calculated with the σ–τ method, without invoking random selections of observations. The approach to the calculation of CC_1/2_ has a number of advantages compared with the random-selection method using the formula for Pearson’s correlation coefficient:

(*a*) It avoids the numerical spread of results associated with different seeds of the random split assignment and is therefore more accurate.

(*b*) It treats odd numbers of reflections consistently, which otherwise lead to unequal numbers in the two half-datasets, which again leads to more accurate results.

(*c*) It treats the sigma weighting of merged intensities more consistently. The original derivation of properties of CC_1/2_ (Karplus & Diederichs, 2012 ▸, supplement) does not take weighting of intensities into account, whereas our formulation in §2.3 naturally accommodates weighted intensities.

(*d*) The higher precision of CC_1/2_ values allows us to calculate precise ΔCC_1/2_ values that would vanish in the noise incurred by random assignments.

(*e*) The calculation of the anomalous CC_1/2_ (CC_1/2_ano_) can be done analogously. For CC_1/2_ano_, the formula suggests an answer to a question that has puzzled several crystallographers and was discussed on the CCP4 bulletin board (CCP4 Bulletin Board, 2015 ▸): why do we sometimes see negative (mostly anomalous) CC_1/2_ values in high-resolution shells? The formula tells us immediately that this symptom indicates that the average variance of the observations is higher than the variance of the averaged intensities in those particular resolution shells. Obviously, this is consistent with the given situation in which practically no anomalous signal but the usual measurement error is present.

### 4.2. Choice of target function and rejection criterion   

There exist two types of errors in crystallographic intensity data: random and systematic. If only random errors are present in the observed intensities *I_i_*, no datasets should be discarded, no matter how weak they are, since they improve the merged intensities *I*_merged_ if the 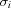 are derived from counting statistics. This situation defines ‘isomorphism of datasets’, in which the following hold:

(i) The relations 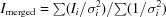 and 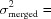 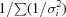 hold strictly, and therefore 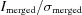 grows monotonically if more data are merged.

(ii) 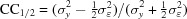 (as defined in §2) also grows monotonically since the mean error decreases if more data are merged. The value of 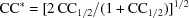, itself monotonically depending on CC_1/2_, then is an accurate indicator for the correlation of the merged data and the (unknown) true data (Karplus & Diederichs, 2012 ▸).

However, if systematic errors exist – which is unfortunately always the case, to some extent – these are by definition not independent. The above relations for 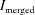, 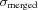 and CC_1/2_ then tell us the precision, but not the accuracy, of the merged intensities.

The 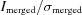 ratio still increases in the presence of systematic errors, since the denominator of 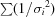 grows with every observation merged. 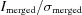 is therefore not suitable for identifying systematic error. CC_1/2_, on the other hand, diminishes if data with a sufficient amount of systematic error are merged, since it depends on the agreement of the observed intensity values. Non-isomorphism between datasets is a special case of systematic errors which affect all reflections in a dataset in a way that may in principle be different for every dataset. For simplicity, however, we may assume that most datasets do not differ significantly in systematic ways. This assumption is valid if the crystals are grown from the same protein preparation under the same conditions, the crystals are mounted, measured and processed in the same (or a closely similar) way, and indexing ambiguities (if applicable) have been resolved (Brehm & Diederichs, 2014 ▸).

A single rogue (outlier) dataset then has a small influence on the merged data, but if even the small part of the total dataset that it influences leads to a significant decrease of CC_1/2_, this may be considered a strong hint towards non-isomorphism of this particular dataset, and it appears justified to discard it. Its exclusion should reduce the noise in electron density maps and improve the agreement between merged data and the refined model.

A small degree of non-isomorphism in a dataset may still allow a slight increase or lead to an insignificant decrease of CC_1/2_, such that this dataset cannot be identified with the ΔCC_1/2_ method. If a large number of such datasets are merged, this will lead to a degradation of the merged intensities, because they introduce into the merged data a mixture of signals corresponding to molecular conformations or states distant from the majority one. Refinement of a single model against such merged data will ultimately also result in elevated *R*_work_/*R*_free_ and noise in electron density maps. This means that many slightly non-isomorphous datasets may result in a slight increase of CC_1/2_ while nevertheless decreasing the suitability of the data for refinement.

An increase of CC_1/2_ is thus a necessary but, because of this caveat, not strictly a sufficient condition for improvement of data by merging. In principle, the ΔCC_1/2_ method shares this restriction with the BLEND method (Foadi *et al.*, 2013 ▸), which uses a large cell parameter deviation as rejection criterion. However, since it uses the experimental intensity data, the ΔCC_1/2_ method directly targets the desired property of optimizing the merged intensity data, and is successful in doing so as seen when being validated. Compared to the pairwise-correlation method of Giordano *et al.* (2012 ▸), which interprets low correlation as meaning low non-isomorphism, we argue that our method avoids the erroneous rejection of weak datasets, at least in situations where the majority of datasets are isomorphous and a mixture of strong and weak datasets exists.

### 4.3. Non-isomorphism in simulated and experimental data   

If datasets are artificially modified such that non-isomorphism is introduced by increasing amounts of unit-cell inflation, a direct relation between ΔCC_1/2_*i*_ and the amount of unit-cell change is found (Table 2 ▸). We find that changes in the unit cell from 0.4 Å can be considered as non-isomorphous for this combination of datasets. This does not mean that non-isomorphism caused by unit-cell changes is in general not detectable below 0.4 Å; in fact the threshold is dependent on the resolution of the data and the specific combination of datasets, which is why we propose an iterative usage of the method.

The most isomorphous dataset is the one with the average of all unit-cell dimensions, which appears to confirm the method of Foadi *et al.* (2013 ▸). The latter method would not have been of much help for the PepT and AlgE projects, however, because their partial datasets have poorly determined unit-cell parameters and therefore our *a posteriori* analysis could not reveal any particular unit-cell-related deviations or properties of non-isomorphous datasets.

For the latter projects, identification of non-isomorphous datasets was straightforward with the ΔCC_1/2_ method. Owing to our precise method for CC_1/2_ calculation, outliers may yield high significance levels, and we expect this to hold also for a larger number of datasets.

Unfortunately, from a theoretical point of view it remains unclear which properties the outlier datasets have such that they strongly influence the merged data; further work in this area is underway.

### 4.4. Pitfalls of validation   

One way of validating the identification of non-isomorphous datasets would be to refine a model against merged data with and without the dataset in question and to compare *R*_work_/*R*_free_ of the two refinements. However, trials to do so convinced us that the small number of free-set reflections present in each partial dataset lead to inconclusive results as *R*_free_ showed large variations.

Likewise, direct comparison of squared structure factors from the model with intensities of partial datasets did not lead to conclusive results, since weak datasets displayed low correlations.

We therefore compared, without refinement, ΔCC_FOC_*i*_, the change in correlation coefficient between observed structure factors and structure factors calculated from their PDB models, with ΔCC_1/2_*i*_. In the case of PepT, we found that those datasets which were identified as non-isomorphous according to ΔCC_1/2_*i*_ also reduce the correlation of the merged data with the model, and thus we confirmed our decision based on ΔCC_1/2_*i*_. However, the AlgE datasets displayed the opposite effect, which puzzled us until we realized that the model we were basing the comparison upon had – in contrast to the PepT model we used – originally been refined against the very data we were comparing it with. In other words, the model had been influenced by all datasets, including non-isomorphous ones, and was therefore biased: exclusion of non-isomorphous data from the merged data resulted in an increase of ΔCC_FOC_*i*_. The remedy we found and employed was to add large random shifts with zero mean to the coordinates of the model, thus reducing the bias by forcing the model out of its biased local energy minimum, at the expense of an overall degraded model with little discriminatory power.

One way of avoiding the bias problem would be to perform refinements of a mildly shaken model and leave out each dataset in turn, to arrive at more realistic unbiased ΔCC_FOC_*i*_ values. However, this would be a project on its own and was considered outside the scope of this work.

### 4.5. Summary   

Our findings demonstrate that non-isomorphous datasets can be identified with an algorithm which uses the numerical value of the average CC_1/2_ across all resolution shells as an optimization criterion. This not only works well with artificial data simulating cell parameter variation but is also demonstrated and validated with experimental data.

Although CC_1/2_ was devised as a precision indicator for the merged data, it can also serve as a proxy for the quality of the model that can be derived from the data, since CC_1/2_ constitutes the link between data and model quality (Karplus & Diederichs, 2012 ▸). This role as proxy is compromised if model bias plays a role. However, contrary to classical model bias where the phases and therefore the electron density map are influenced by the very model that is the goal of structure solution, here we experience a different kind of bias: a dataset influences the model to such an extent that the correlation of model and data always diminishes if that dataset is removed from the merged data (and is strong enough). This leads to the insight that each and every dataset noticeably influences the model, and consequently the model will have to account for all possible constituents and conformations present in the data.

However, systematic differences between crystals cannot properly be modelled in refinement since in serial crystallography the averaging of datasets is incoherently done on intensities, rather than on structure factors as would happen if different conformations occur in the coherently illuminated volume of a single crystal. Since a refined model, with its coherently diffracting constituents, cannot fully approximate a sum of intensities (*i.e.* squared amplitudes) with a (squared) sum of amplitudes, an elevated level of *R*_work_/*R*_free_ and noise in electron density maps would result if non-isomorphism is not detected and the worst datasets are not excluded.

In our trials with experimental data measured at a synchrotron, the outcome was the rejection of only a single (as for PepT and AlgE) or a few rogue datasets. However, this rather attests to the consistent quality of the data obtained in SSX. Finally, we note that our procedure is equally applicable to datasets obtained from SFX. Rejection rates may be higher in the latter method since its technology is less mature than that of SSX and the absolute numbers of datasets are high. We therefore expect that the ΔCC_1/2_ method will be useful in SFX.

## Figures and Tables

**Table 1:** Crystallographic statistics of experimental datasets

	PepT	AlgE
PDB code	4xni	4xnk
Space group	(20) *C*222_1_	(19) *P*2_1_2_1_2_1_
Unit-cell parameters (Å)	*a* = 106.88, *b* = 106.88, *c* = 111.14	*a* = 48.01, *b* = 74.34, *c* = 184.69
Wavelength (Å)	0.979180	1.033000
No. of crystals	159	266
Resolution (Å)	50–2.78	50–2.54
Completeness (%)	97.6	84.7
Completeness highest resolution shell	67.6 (2.85–2.78)	7.6 (2.61–2.54)
Total No. of observations	905 207	151 228
No. of observations per crystal (min–max, mean)	2592–6040, 5693	59–507, 564
No. of unique reflections	16 485	18 684
*R*_meas_	0.973	0.565
CC_1/2_	0.992	0.926
〈*I*/σ(*I*)〉	4.25	2.74

**Table 2:** ΔCC_1/2_*i*_ of synthetic datasets with elongated unit-cell parameters

Change of unit-cell parameters (Å)	ΔCC_1/2_*i*_
+1.0	−0.518
+0.8	−0.313
+0.6	−0.271
+0.4	−0.262
+0.2	+0.873
+0.1 (2 datasets)	+0.785, +0.785
0.0 (4 datasets)	+0.710, +0.706, +0.698, +0.684
